# Automated detection of celiac disease using Machine Learning Algorithms

**DOI:** 10.1038/s41598-022-07199-z

**Published:** 2022-03-08

**Authors:** Cristian-Andrei Stoleru, Eva H. Dulf, Lidia Ciobanu

**Affiliations:** 1 Automation Department, Faculty of Automation and Computer Science, Technical University of Cluj-Napoca, Memorandumului 28, 400014 Cluj-Napoca, Romania; 2 Faculty of Medicine, Regional Institute of Gastroenterology and Hepatology, Iuliu Hatieganu University of Medicine and Pharmacy, Croitorilor Street 19-21, 400162 Cluj-Napoca, Romania

**Keywords:** Gastrointestinal diseases, Biomedical engineering

## Abstract

Celiac disease is a disorder of the immune system that mainly affects the small intestine but can also affect the skeletal system. The diagnosis relies on histological assessment of duodenal biopsies acquired by upper digestive endoscopy. Immunological tests involve collecting a blood sample to detect if the antibodies have been produced in the body. Endoscopy is invasive and histology is time-consuming. In recent years there have been various algorithms that use artificial intelligence (AI) and neural convolutions (CNN, Convolutional Neural Network) to process images from capsule endoscopy, a non-invasive endoscopy approach, that provides magnified, high qualitative images of the small bowel mucosa, to quickly establish a diagnosis. The proposed innovative approach do not use complex learning algorithms, instead it find some artefacts in the endoscopies using kernels and use classified machine learning algorithms. Each used artefacts have a psychical meaning: atrophies of the mucosa with a visible submucosal vascular pattern; the presence of cracks (depressions) that have an appearance similar to that of dry land; reduction or complete loss of folds in the duodenum; the presence of a submerged appearance at the Kerckring folds and a low number of villi. The results obtained for video capsule endoscopy images processing reveal an accuracy of 94.1% and F1 score of 94%, which is competitive with other complex algorithms. The main goal of the present research was to demonstrate that computer-aided diagnosis of celiac disease is possible even without the use of very complex algorithms, which require expensive hardware and a lot of processing time. The use of the proposed automated images processing acquired noninvasively by capsule endoscopy would be assistive in detecting the subtle presence of villous atrophy not evident by visual inspection. It may also be useful to assess the degree of improvement of celiac. Patients on a gluten-free diet, the main treatment method for stopping the autoimmune process and improving the state of the small intestinal villi. The novelty of the work is that the algorithm uses two modified filters to properly analyse the intestine wall texture. It is proved that using the right filters, the proper diagnostic can be obtained by image processing, without the use of a complicated machine learning algorithm.

## Introduction

The Celiac disease (which origins came from the Greek word, Koiliakos, which means abdomen) is a disorder of the immune system that mainly affects the small intestine with systemic complications^1^. The disease is caused by a reaction to gluten, a diverse group of proteins found in wheat, barley, and rye^1^. Exposure of patients to this protein produces an immune reaction that affect various organs, but the most severe effects are present in the small intestine, where they cause a flammable reaction that leads to a decrease in villi (prominent cylindrical or conical formations that have the role of absorbing digestive products), which causes malabsorption, weight loss and diarrhoea^2^.

Small intestinal biopsy is the mainly used diagnostic procedure. New approaches include highly sensitive and specific serological tests, such as tissue transglutaminase, endomysial and deamidated gliadin peptide antibodies^2^. The most diagnosis relies on histological assessment of duodenal biopsies, obtained by upper digestive endoscopy. In advanced forms of the disease the endoscopic image of duodenal mucosa is highly suggestive of celiac disease. Immunological blood tests might help the diagnosis and are frequently used for screening the populations at risk. Genetic tests are required to screen the family members for predisposition if one member is affected. Antibody tests often come out as false negative or false positive, especially in people with immunoglobulin A deficiency, type 1 diabetes^3^, hepatitis, Hashimoto’s thyroid, and rheumatoid arthritis. Endoscopies can be misinterpreted since the villi are slightly affected and the intestines do not show differences.

Endoscopy is the procedure performed by a medical professional to be able to see inside the human body. It uses an endoscope (tubular instrument, optical lighting that transmits information through cameras) that is inserted into a hole in the body to analyse, in general, the digestive system^1^.

In the case of celiac disease, the upper digestive endoscopy is mandatory, as it enables duodenal biopsy. Different endoscopic techniques, such as water immersion, dye-based chromoendoscopy, and virtual chromoendoscopy (narrow band imaging) are used in order to better depict small changes of duodenal mucosa suggestive of incipient celiac disease^1^. But the endoscopy is invasive for patients. In order to make a more rapid and comfortable diagnosis, capsule endoscopy, a non-invasive device is used to acquire magnified, high qualitative images from small bowel.

To better understand the effects of these diseases and the role of endoscopy, it is necessary to consider the structure of the small intestine. It consists of three main parts: the duodenum, jejunum, and ileum. In general, in the case of classical endoscopies performed to detect this condition, an analysis is done in the first two parts of the duodenum^4^. The capsule endoscopy allows the visualization of the whole small bowel mucosa.

To reduce the training time of medical staff, in recent years there have been various algorithms that use artificial intelligence (AI, Artificial Intelligence) and neural convolutions (CNN, Convolutional Neural Network) to process images from endoscopes, to quickly establish a diagnosis. The problem that appears in most computer tests is that they cannot analyze a response that comes from a different endoscopy than those used to train algorithms.

Computer diagnosis system is gaining interest in recent studies. The paper^5^ presents an algorithm based on images from the biopsy resulting from an endoscopy and uses an algorithm based on neural networks, but due to the diversity of endoscopes and the fact that some symptoms of celiac disease are local, not a single captured area represented by an image. The algorithm will not give good results on a much larger set of data. In Ref.^6^ is presented a different approach to the problem, the authors indicating the percentage chances that the patient will suffer from celiac disease. But due to the small number of data used (even if a video contains about 600 frames, which means that the analysis uses 6600 pictures for the test part) the fact that only 11 patients were used to determine this model leads to a lack of data diversity. In Ref.^7^ the approach is based on the analysis of light intensity, texture, and the dominant period. It is obtained a model with a high sensitivity that can perform a detection without using complex image processing algorithms. The advantages of using such an algorithm are found in the processing speed of the input data but also in the control over the way they are processed. It is concluded that this analysis and endoscopic image processing method is much more efficient than other existing methods. However, some elements could be improved in such an analysis, for example, the number of folds present in the footage. In several papers are discussed ways to use deep learning algorithms to approach that distinguishes between Celiac Disease (CD) and Environmental Enteropathy (EE) and normal tissue from digitized duodenal biopsies. The authors of paper^8^ uses the predefined ResNet50 architecture to classify their patches. In work^9^ is trained a model based on deep residual networks to establish a histological scoring system called the modified Marsh score. Paper^10^ proposed a diagnostic system using a series of predictive models based on statistical approaches: logistic regression, elastic net, tree-based models, support vector machine, neural network, random forest and linear discriminant analysis. All these experimental results show accuracies over 90% but uses complex algorithms for processing. In Ref.^11^ are discussed four machine learning methods to select features predictive for Celiac disease: Random Forests, Extremely Randomized Trees, Boosted Trees, Logistic Regression. Although powerful methods are used, the accuracy are only above 75%. In work^12^ are used selective machine-learning techniques for diagnosis. The added value of the paper lies in scrutinizing the possible effects that the presence of Type 1 Diabetes, Type 2 Diabetes, Autoimmune Thyroid Disease, and Non Autoimmune Thyroid Disease have on the occurrence of Celiac disease. In Ref.^13^ is presented a simple method of diagnosing the patient using image-based analysis. The images were divided into several sub-images to perform an analysis based on the variation of contrasts and their texture. The research led to a threshold classifier with 80% sensitivity and 96% specificity. In research^14^ the authors conducted a study which combines several algorithms in this field. Performing independent extractions, they evaluated the methodological quality of each study, establishing the relevance of the data acquisition in connection with the desired diagnosis. Based on these data, several models have been developed to predict whether an image to be analysed come from a person with celiac disease or a healthy person. These models were later described based on certain parameters such as sensitivity, probability ratios, and diagnostic probability, followed by a description of possible complications and the costs generated by them. Three of the analysed algorithms had a sensitivity higher than 80% if they did not report major complications, which led to a low cost. This paper presents new variables that must be considered in research in this field, being related to the possibility of a false diagnosis, and the costs it raises. The conclusion made by the researchers at the end of the paper indicates that there is a possibility that in the future this diagnosis can be given by a computer, but still needs a broader analysis in this area, both on the algorithmic and safety side. A more advanced approach is presented in the paper^15^, where a “Random Multimodel Deep Learning, RMDL” architecture is used to process the information obtained from the analysis of images from the endoscope. This method combines various architectures and structures of Deep Learning. The final answer is given by a majority vote. The results lead to an accuracy of 93%. This approach requires also intestine biopsy for microscope image processing. This operation is much more difficult and invasive than a simple endoscopy and requires the presence of an experienced medical staff. The algorithm has the role to validate the doctor’s decision.

However, a diagnosis system based on simple endoscope image analysis is a better solution for both clinician and patient if ensures high accuracy. This is the goal of the present research.

The novelty of the method presented in this paper lies in the used approach. Are used two modified filters, one modified Sobel filter, and one special one, created especially for these images. After these filter applications, the algorithm will adjust the images to analyse the intestine wall texture. The main idea is that by using the right filters, we won’t need a complicated machine learning algorithm to make a diagnostic for the celiac disease. For a proper diagnosis, the system requires five images from each patient from the following regions: bulb, duodenum, jejunum, ileum, and ileum-distal. For a time and hardware saving procedure are not used complex learning algorithms, instead are find some artefacts in the endoscopies using kernels and with them, a classified Machine learning algorithm is trained. The artefacts searched by the proposed diagnostic system are:

- atrophies of the mucosa with a visible submucosal vascular pattern;
- the presence of cracks (depressions) that have an appearance similar to that of dry land;
- reduction or complete loss of folds in the duodenum;
- the presence of a submerged appearance at the Kerckring folds;
- Low number of villi.

The structure of the paper is the following. After this short Introduction, “Introduction” section presents the data acquisition details, while “Methods” section details the proposed video processing method. “Results” section discuss the numerical data classification. “Conclusion” section presents the results. The work ends with concluding remarks.

## Methods

**Figure 1:**
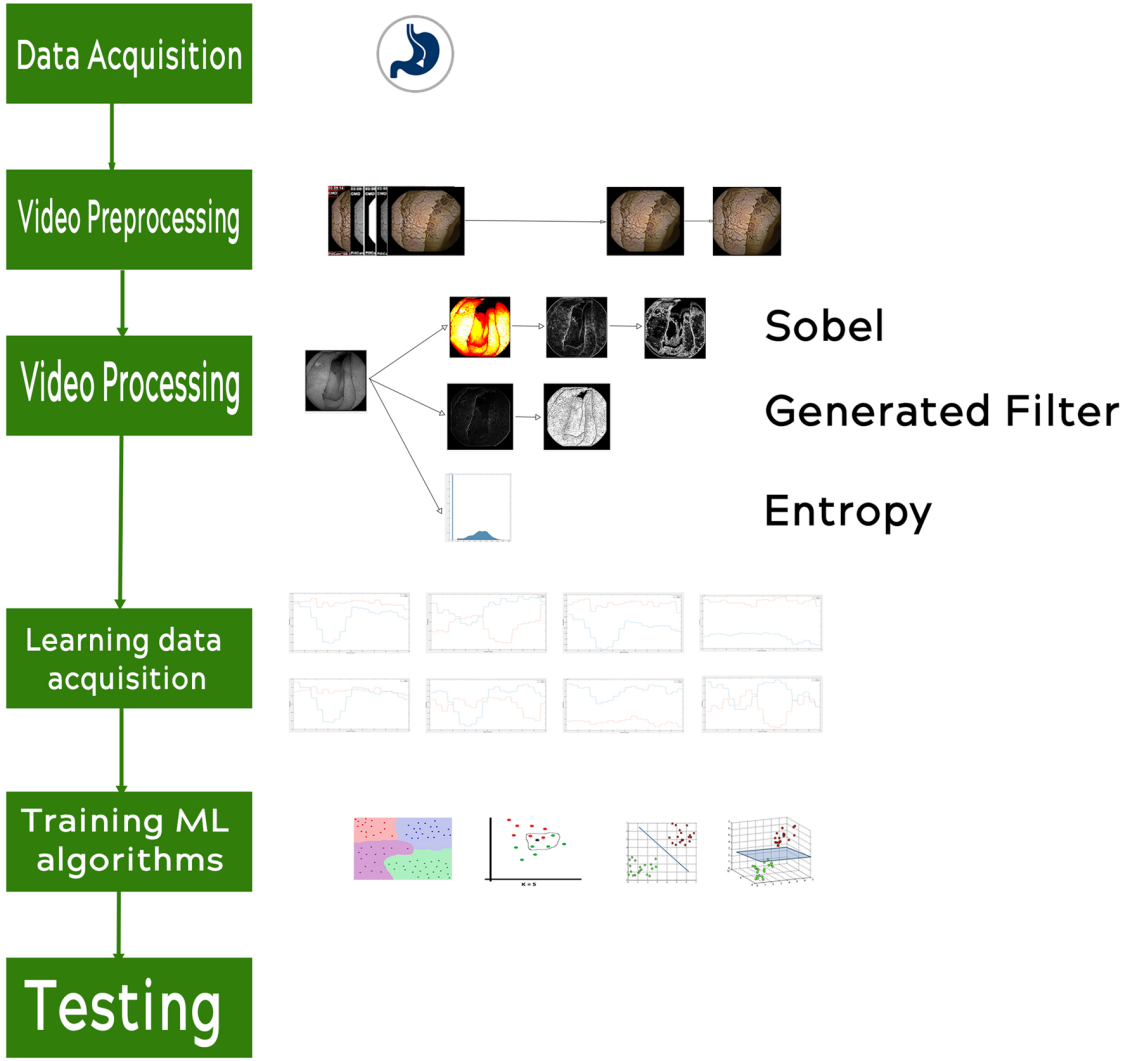
Experiment workflow.

The workflow of the research is presented in Fig. 1.

### Dataset

The first step in developing a machine learning algorithm is to obtain a set of data from the information from an endoscope. These were obtained following the application of operations and extractions that will be presented in the following chapters.

The data set consists of 109 films (of 100 frames), of which 45 come from healthy patients and 65 come from patients suffering from celiac disease. The classification of these videos was made by “Iuliu Hatieganu” University of Medicine and Pharmacy after using a PillCam SB3 gastroscopy device throughout the year 2019, with patients of different ages and genders (63.3% of patients are older than 35 and 54.1% were man). These data were divided into three categories, as follows:

- 51 videos, used for the training data set.
- 51 videos, used for the test set.
- 7 videos, kept for real-time testing of the application.

A computing machine using a 2.3 GHz quad-core processor capable of running 8 threads in parallel (AMD Ryzen 7 3750H), a dedicated video card with 6 GB of memory, was used to process the data, with a processing speed of 1140 MHz and a memory bandwidth of 192 bits (Nvidia GeForce 1660Ti Max-Q), and volatile memory (RAM) of 8 GB.

The final dataset is made of 105 videos. During the learning process, 4 videos have been excluded because the final data set was normalized, and it was divided randomly so that in the final there were approximately 50% of the celiac endoscopes and 50% of the healthy ones in the training set.

### Video processing

Each video obtained from the endoscope contains 100 “frames” (images) that have been analysed and processed to discover data that can be correlated with the diseases presented above. The videos from endoscopy are preprocessed in images where the information about the medical equipment is deleted, and the images are then cropped to optimize the time needed to analyse them, thus the video processing problem is at the same time an image processing problem that must standardize a general algorithm used for each frame. Extensive data augmentations were performed to prevent overfitting the model. Both rotational and axial symmetry, mirroring and zoom were used, as recommended in papers^11–16^. In the following paragraphs, it will be described the algorithms used on the images.

#### Detection of spectral variation

The human eye can easily see the difference in the variation of light spectra in healthy and sick patients. To be able to create an algorithm that can perceive the information that the human eye transmits to the brain, it is needed to understand how an image is interpreted by the computer. In reality, there are two types of images, black and white, and colour. These are interpreted differently by a computing machine by the way it stores and displays them. Suppose a black and white image has a resolution of M × N pixels (for example, an HD image has a resolution of 1920 × 1080), where each pixel is stored in computer memory by a byte, whence it results that each image is saved in a matrix of M columns and N rows. The value in the byte can be between 0 and 255 and represents a shade of grey that describes the light intensity. Things are a little different when it comes to a colour image, which is saved in the computer’s memory, depending on how the visual spectrum is interpreted. The most common is the one interpreted by the human eye, the one formed by the colours red, green, and blue (RGB), but in certain situations, CYMK (cyan, magenta, yellow and black) can also be used. In this paper, it will be used the RGB spectrum, which is saved in computer memory through three matrixes, each representing a colour in the spectrum, which superimposed obtain the colour image.

#### Eliminate unwanted information

**Figure 2:**
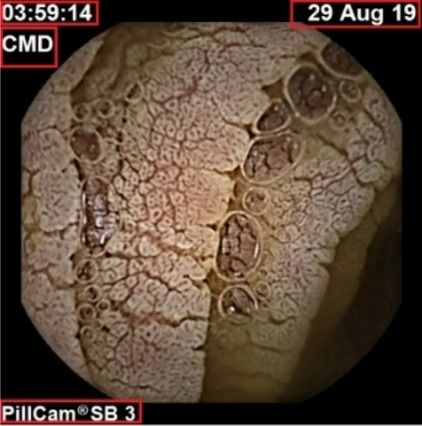
Unwanted information.

Each frame in the endoscopes contains information about the date and time they took place, the source of acquisition, and the device used to process them. These elements are highlighted in Fig. 2.

**Figure 3:**
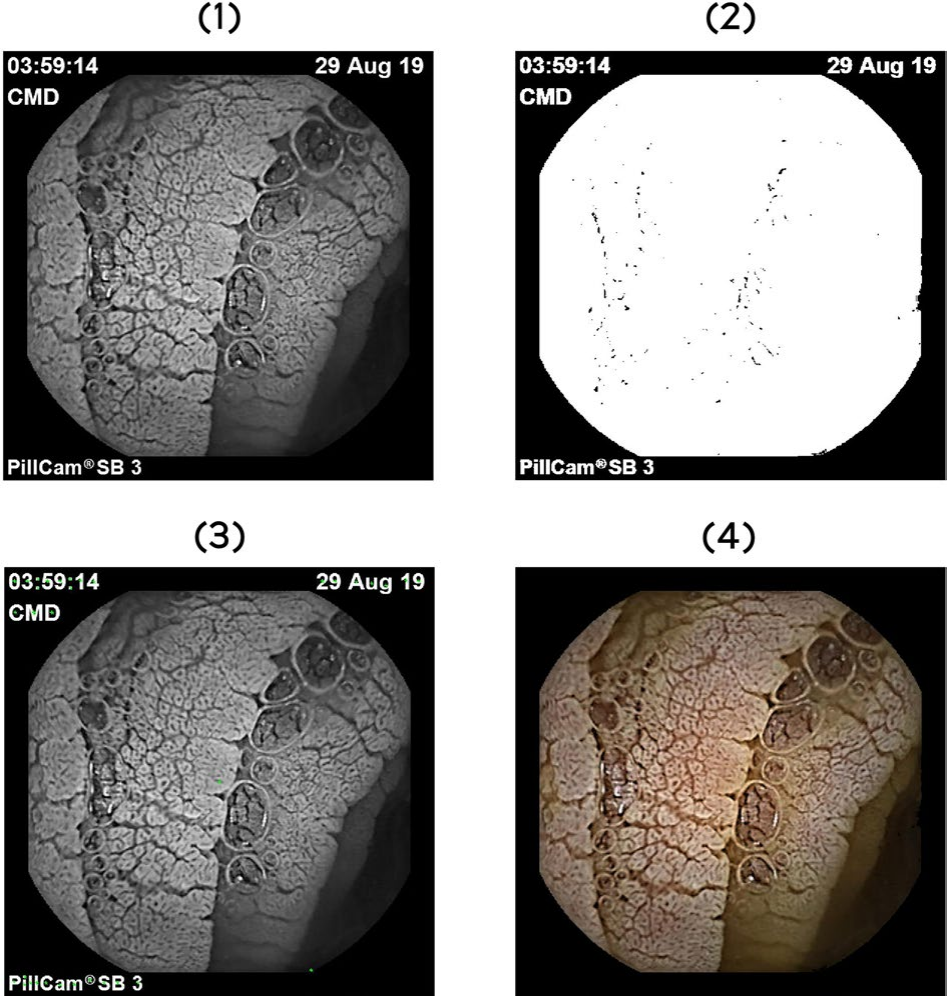
Steps required to remove the unwanted information. **(1)** Black and White image, **(2)** binary image, **(3)** centre of the regions, **(4)** final result.

The first step in removing the information is to turn this image into a black and white image which is binarized to obtain the mask of elements other than black (the value 0 in indices of grey intensities). Following this process, several regions will be obtained (white areas delimited by black) that will be approximated with the coordinate in their centre so that the regions containing text will be found in the edges of the image, and the elements in endoscopy will be in the centre. The regions that will be saved in the new image will have the centres outside the border of 20% of the image. An example of this algorithm results is presented in Fig. 3.

#### Cropping images

**Figure 4:**
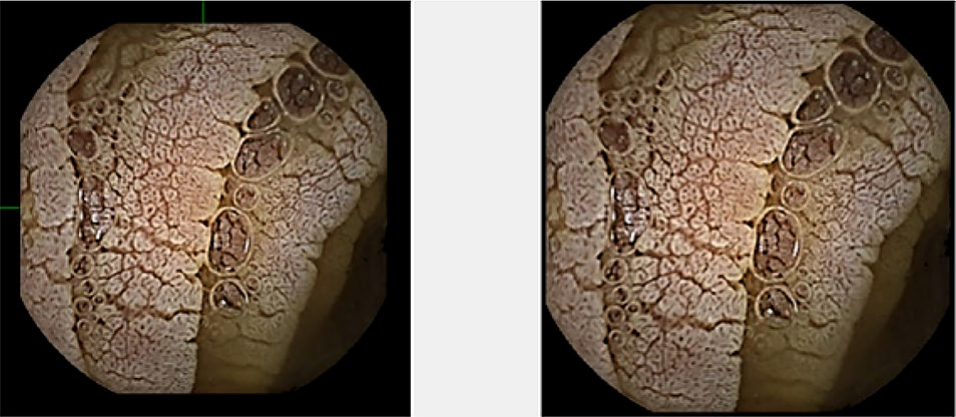
Before and after the cropping.

To reduce the processing time of the images, it is needed to reduce their size depending on the black border present in the endoscopes. The chosen algorithm is based on traversing the image matrix in width and length until it meets a value other than 0 (the value of the colour black in the spectrum of grey intensities). When meeting that value, it will retain its position and will crop the image in height and width depending on the number of pixels obtained. An example of result is represented in Fig. 4. Even if the time required for a current image processing do not seem large, this time multiplies depending on the number of images in the video, so this step is mandatory.

#### Region detection

During the analysis of the footage from the endoscope, it is noticeable a major difference in the number of regions from a patient suffering from celiac disease to a healthy person. These regions are obtained by applying the two—vertical and horizontal—Sobel filters.

The Sobel filter measures the 2-D spatial gradient on an image and thus highlights the regions with the high spatial frequency that corresponds to the edges. It is usually used to find the magnitude of the absolute gradient in every point of a black and white image. In theory, the operator consists of a pair of 3 × 3 convolution cores as shown in Table 1. The second core is simply the first core rotated 90 degrees^16^.

**Table 1:** Sobel filters.

Horizontally Sobel			Vertical Sobel		
+ 1	0	− 1	+ 1	+ 2	+ 1
+ 2	0	− 2	0	0	0
+ 1	0	− 1	− 1	− 2	− 1

To obtain both the vertically and horizontally contour, are approximated the results of the two convolutions according to Eq. (1):

1 $${G}_{xy}=\sqrt{{G}_{x}^{2}+{G}_{y}^{2}},$$

where Gx is the result obtained after the application of the vertical core, Gy is the result obtained after the application of the horizontal core^16^.

**Figure 5:**
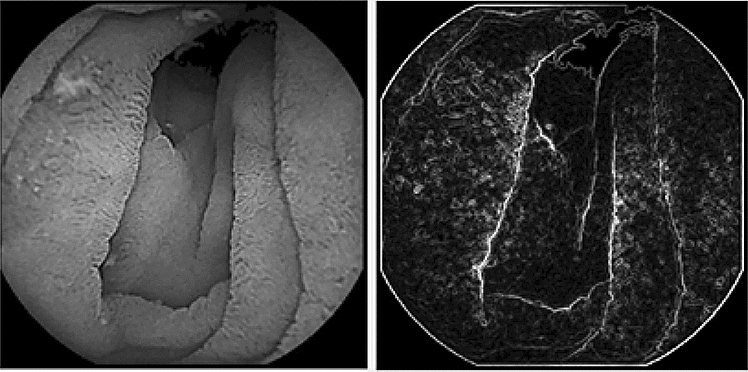
Before and after Sobel filter.

Figure 5 shows the result of applying the Sobel filter using these convolution operations. Due to the excess information on the intestinal walls, some image transformations are needed to get only the outline of the walls.

To reduce the amount of information found after applying the Sobel filters, it is necessary to change the brightness of the image, achieving this by changing the contrast based on the average values of pixel intensity and their standard deviation. Thus, each pixel in the image is brought to a contrast value between the limits given by the Eq. (2):

2 $$\left\{\begin{array}{c}Mean-0.2\times Standar{d\, deviation}\\ Mean+0.2\times Standar{d \,deviation}\end{array}\right..$$

**Figure 6:**
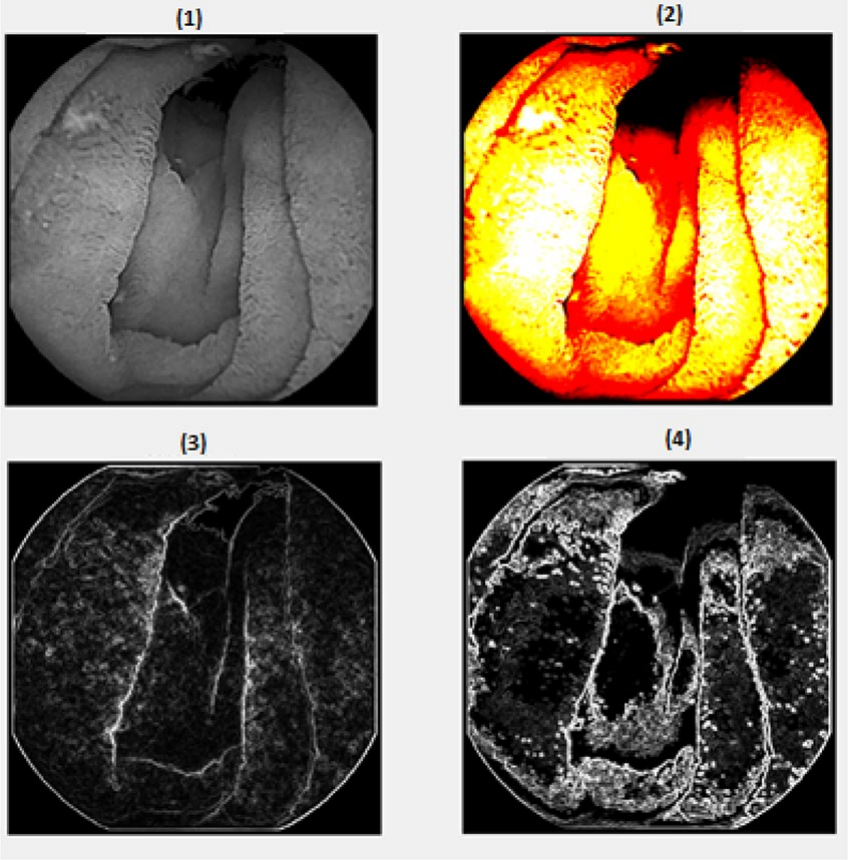
Modification to the Sobel filter. **(1)** Black and white Image, **(2)** contrast adjustments, **(3)** simple Sobel filter, **(4)** final result.

The result is presented in Fig. 6, where it can be observed that the edges of the intestinal segments are delimited much better than in the classical approach and the information found on the walls of the digestive system is reduced to the main information. With this method the final application will calculate through the variation of wrinkles, which is used in obtaining a correct diagnosis for celiac disease.

#### Cracks detection

With the decrease of the villi in the intestine (brush-like organic structures), both in size and number cracks like those of dry hands begin to appear, these being, in general, larger in size, which can be easily detected. As in the previous section, this can be done with a filter (also called a kernel), but the problem is that there is no standardization or concrete solution for this.

To solve this problem, it is necessary to know how the convolutions work and generate a filter to help us perform the detection. In theory (according to Eq. (2)), this operation calculates for each pixel in the images a new value, depending on its neighbours^16^.

3 $$Y\left(m,n\right)= \sum_{j=-\infty }^{-\infty } \sum_{i=-\infty }^{-\infty }X\left(j,i\right)\times H(m-i,n-j).$$

The kernel will mark the cracks with a small value, and other components of the intestine with a value above 5, to be able to binarize the image in the future and obtain two areas, the cracks, and the intestine. To simplify the problem, it will use a 3 × 3 core presented in Table 2.

**Table 2:** Theoretical Kernel values.

A ≤ 0	B ≤ 0	C ≤ 0
D ≤ 0	E ≥ 0	D ≤ 0
E ≤ 0	F ≤ 0	G ≤ 0

**Figure 7:**
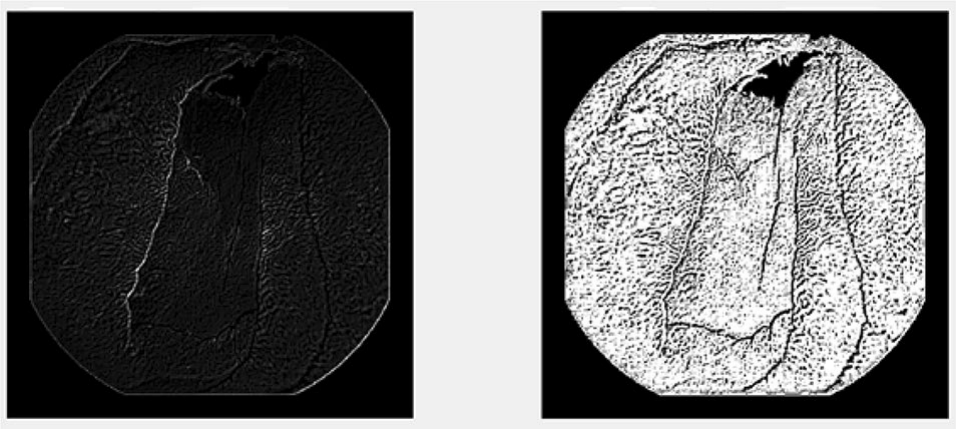
Before and after binarization.

The main idea is to increase the value of the pixel, which is why E is greater than 0, and to decrease the neighbouring values. This is due to the fact that cracks generally have small values, which will lead to a better marking. Through several filter tests, the best result is presented in Fig. 7, where you can see both the image, after applying the filter, and the result obtained after binarization. For this kernel, the following values were used: A =  − 2.3, B =  − 1, C =  − 0.3, D =  − 1.4, E = 7.8, F =  − 1.4; G =  − 0.3, H =  − 1 and I = 0.

#### Entropy analysis

Entropy is defined as the median information of the image and measures the degree of randomness in an image. In more detail, it can be said that it is a lower limit for the bit coding average, per pixel, which can be achieved through an optimal coding scheme without loss of information. It can be used to detect the focus of an image because, in an area of the focused image, the entropy has higher values than in non-focused areas. The entropy calculation is performed using the first-order histogram of the image, which shows the frequency of occurrence (or probability) for all grey levels other than W (or optical density D) in the image^17^.

A histogram is a type of graph that provides a visual interpretation of numerical data, by indicating the number of data points that are in a range of values. These intervals are called classes and the frequency of each is described by a bar, and the higher the frequency, the longer it is. The classes in a histogram of an image represent the intensity of the pixels (or their grey variation)^16^.

**Figure 8:**
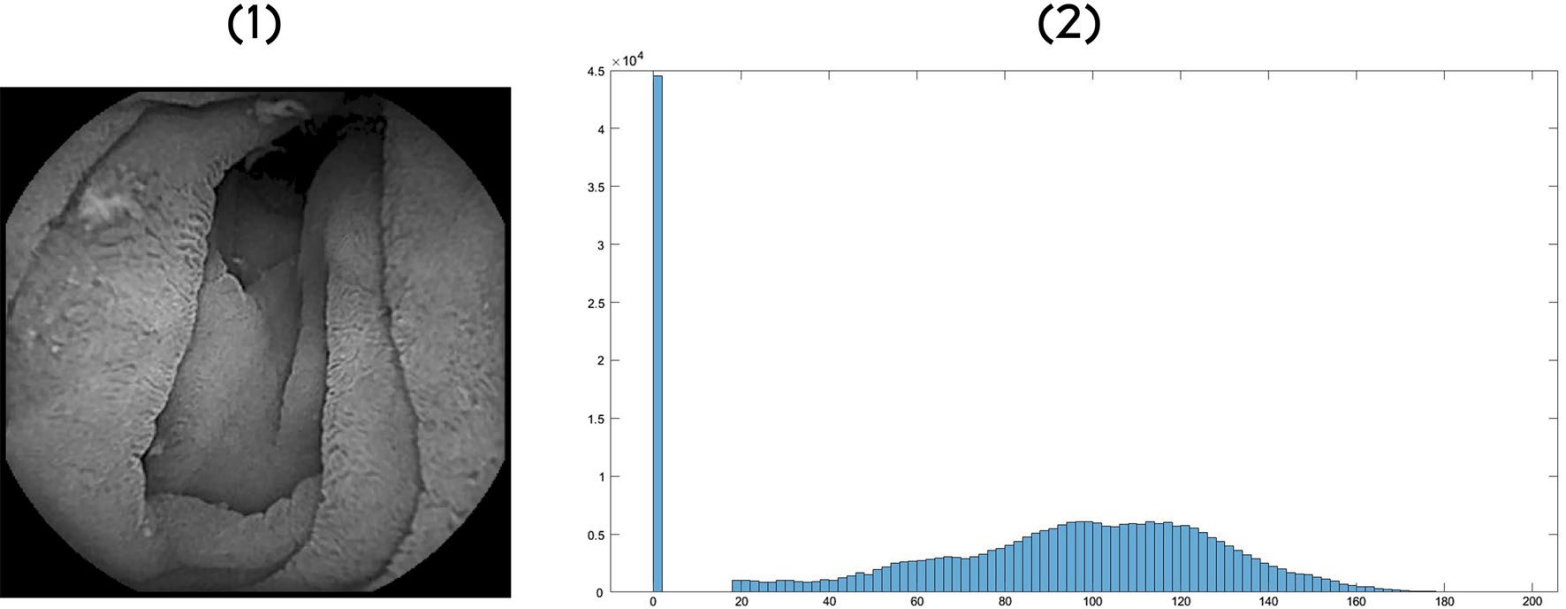
Entropy analysis. (1) Black and White image. (2) Histogram of the image.

The role of entropy in obtaining a diagnosis for celiac disease is that it can also describe the variation of the texture of an image. Figure 8 shows the histogram of an image whose entropy has a value equal to 6.44.

### Classification of numerical data

Numerical data classification is a technique by which data mining can be performed and is used to predict the category to which a data set belongs. The classification is done using a supervised learning technique that is accompanied by the appropriate labels^18^.

Data mining is done using in turn complex techniques of information analysis, by identifying patterns and interactions between the variables of the training data set, to achieve a correct classification for new data (test data)^18^.

In this paper, it is necessary to classify the health of a patient, in one of the two possible situations: healthy or celiac. The data underlying the training of the algorithm comes from the camera of an endoscopic device. Next, it will be presented three algorithms used for the learning problem.

#### KNN (K-nearest neighbor)

This algorithm is one of the simplest, but still one of the most efficient classification algorithms. The basic idea of this algorithm is that similar objects are found at a very short distance from each other^19^.

**Figure 9:**
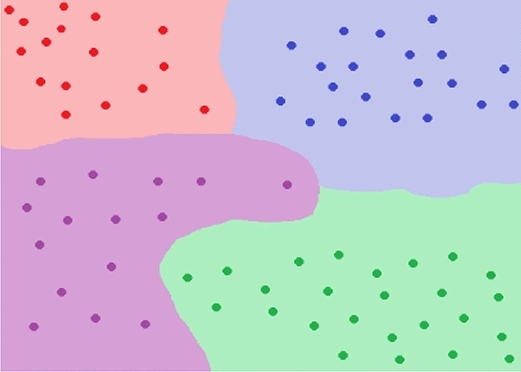
KNN example.

The use of this algorithm requires a data set that can easily distinguish the regions of each data type. Such an example is shown in Fig. 9, where the data are represented by dots, divided by colour, depending on their type. The colour regions on the graph represent locations where if a new data set is introduced, it will be associated with one of the groups, depending on its values.

Another important thing to know about these algorithms is that the K value must be set so that when a new data set is entered, it will look for the nearest K neighbours, and depending on which category is the majority, and the new set will be assigned to that category.

The parameters used in this paper for this algorithm were: number of neighbours = 3 and algorithm was set on auto.

#### Weighted KNN

**Figure 10:**
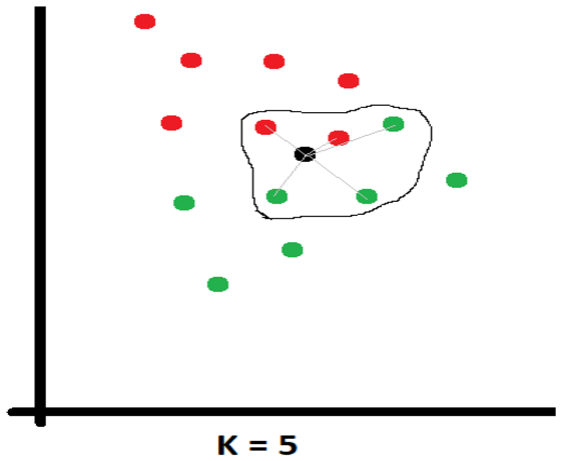
Weighted KNN example.

This algorithm is a variation of the one presented above, where a simple but elegant assumption is made. The impact of the neighbours closest to the new data should be greater than those at a far distance. Figure 10 shows how the neighbours are chosen and how the distance from the black dot to its neighbours is calculated (Euclidean distance). After calculating the distances, it is necessary to choose a function to compute the weight. This function can have various forms, considering each category, but even a simple function such as the inverse of the distance is a good choice. Because the red dots are much closer to the black dot, they have a much higher weight than the sum of the weights of the green dots, which leads to the assignment of the new dot to the red dot, even if the number of green neighbours is much higher.

The parameters used in this paper for this algorithm were: number of neighbours = 3, weight = distance, power parameter was set for Euclidean distance and algorithm was set on auto.

#### Linear SVM

**Figure 11:**
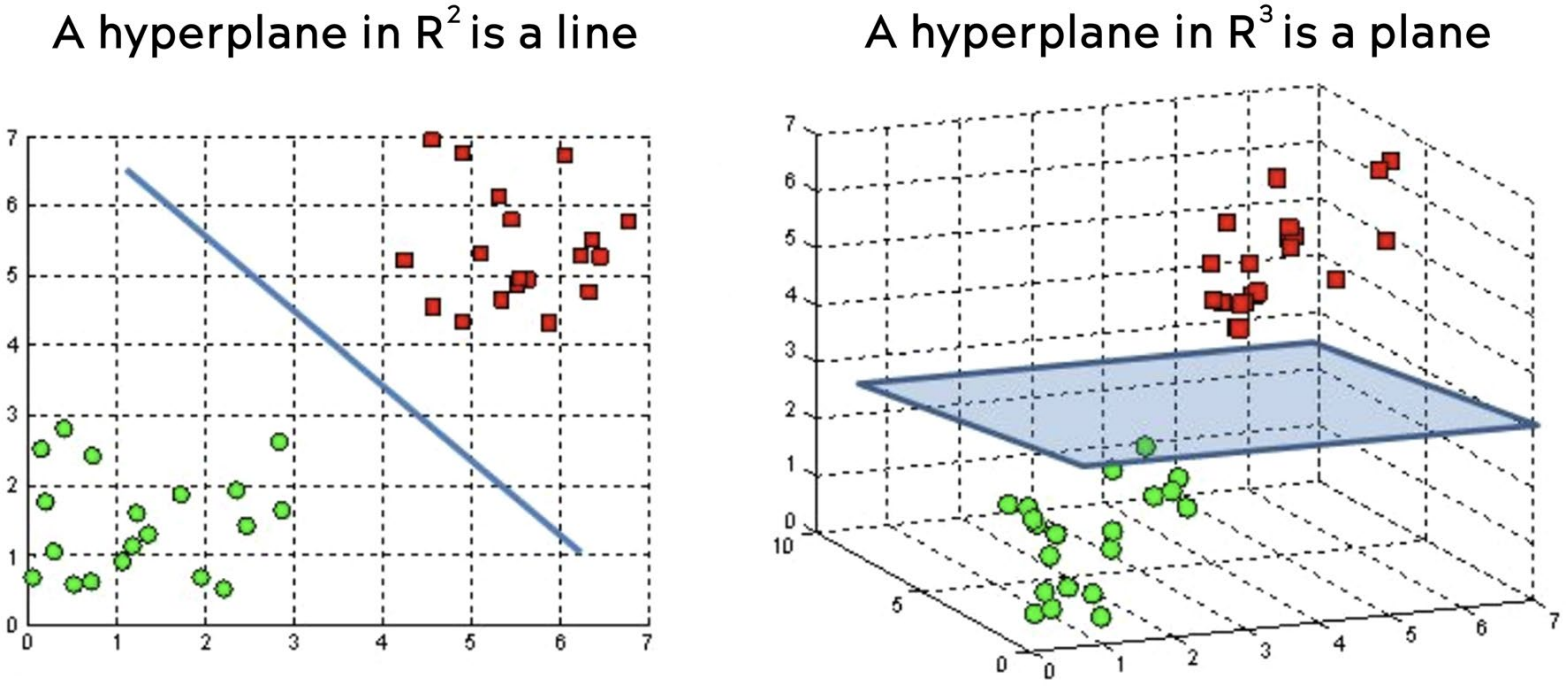
2D and 3D hyperplanes.

This algorithm is part of the SVM (Support Vector Machine) family which are used for both classification and regression operations. The purpose of these algorithms is to find a hyperplane in N dimensions (N being the number of features) that can perform data classification. Figure 11 shows how this algorithm separates the data into two categories (red and green) using a hyperplane (blue). The difference between a linear and a nonlinear algorithm is given by the shape that the hyperplane will have at the end^20^.

The parameters used in this paper for this algorithm were: penalty was set on standard, loss was set on hinge, tolerance was set on 1e−3, intercept was set on true and for class weight was set on balanced and verbose was set on 0.

### Ethics approval and consent

The study was conducted in accordance with the Declaration of Helsinki Ethical Principles and Good Clinical Practices, being approved by the Ethical Committee of Regional Institute of Gastroenterology and Hepatology, Cluj-Napoca, Romania.

## Results

In the first part of this section are presented and discussed the differences from a healthy patient to a patient suffering by celiac disease in terms of numerical data extracted from endoscopes, to understand the role of each artefact. These are:

- The average value of light intensity.
- Standard deviation of the light intensity value.
- Red spectrum variation.
- Green spectrum variation.
- Variation of the blue spectrum.
- The values obtained after applying the Sobel filter.
- The values obtained following the application of the generated filter.
- The value of entropy.
- Variation in the number of large regions.
- Variation in the number of small regions.

In the second part are presented the results obtained from the three neural network algorithms used for classifying data.

### Average variation in light intensity

**Figure 12:**
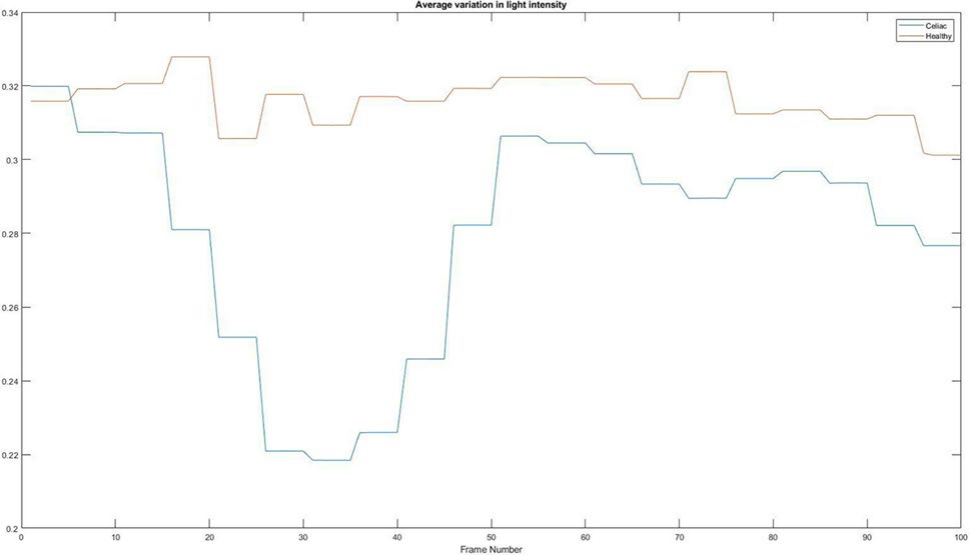
Average variation in light intensity. Red = Healthy and Blue = Celiac.

One of the easiest features to analyse in an image is the amount of light it shows. This shows us the composition of the lining of the intestinal wall and how it reflects light. The first method is to calculate the average value of each image and to analyse its variation on a large number of frames. Figure 12 shows a graph with the different values found from a healthy patient and a patient with celiac disease.

In Table 3 it is presented how the algorithm interprets these values using the mean value, standard deviation, and variation.

**Table 3:** Interpretation of the average variation of light intensity.

Patient	Mean value	Standard deviation	Variation
Healthy	0.3162	0.0063	3.92e−5
Celiac	0.28	0.0301	9.07e−4

### The standard deviation of the light intensity value

**Figure 13:**
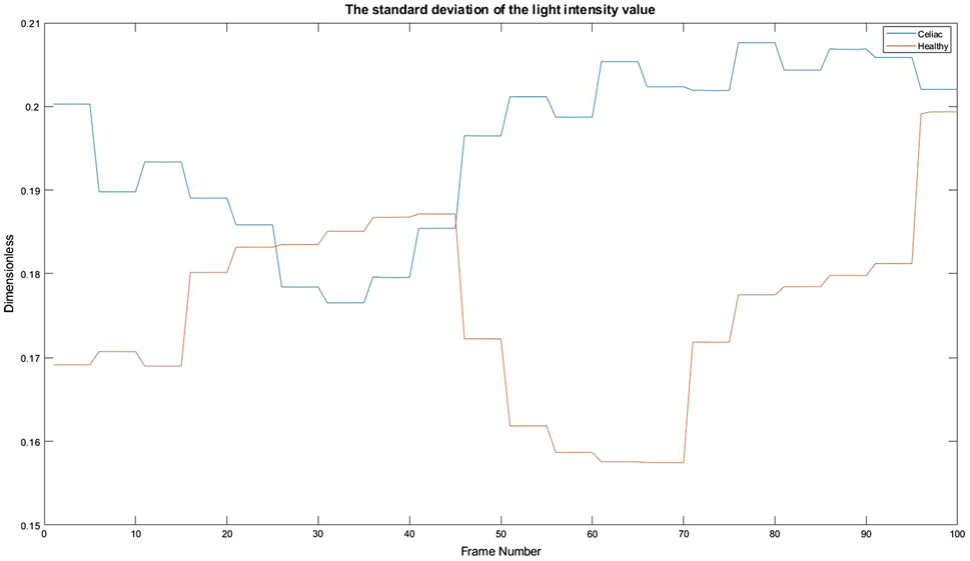
The standard deviation of the light intensity value. Red = Healthy and Blue = Celiac.

The appearance of light in an image can be interpreted by several methods, but in this paper were used only the arithmetic mean of the values, presented in the previous section, and the standard deviation to see an average variation of light intensity in each frame. This can help to detect the presence of cracks or channels in each image. Figure 13 shows by a graph the variation and values obtained for a celiac patient and a healthy one.

In Table 4 it is presented how the algorithm interprets these values using the mean value, standard deviation, and variation.

**Table 4:** Interpretation of the standard deviation of the light intensity value.

Patient	Mean value	Standard deviation	Variation
Healthy	0.1755	0.0109	1.1987e−04
Celiac	0.1956	0.0099	9.7700e−05

### Red spectrum variation

**Figure 14:**
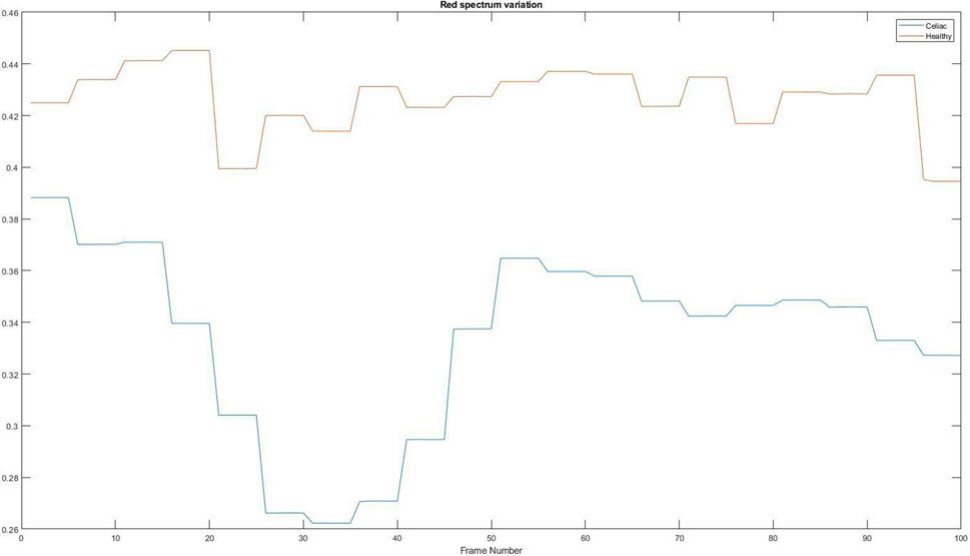
Red spectrum variation. Red = Healthy and Blue = Celiac.

Another factor that can help detect celiac disease is the analysis of each spectrum of light, because, even if it is difficult to see visually, patients suffering from celiac disease have a different shade of colour. Figure 14 shows the variation of the red spectrum with the differences between a patient suffering from celiac disease and a healthy patient.

In Table 5 it is presented how the algorithm interprets these values using the mean value, standard deviation, and variation.

**Table 5:** Interpretation of the average variation of red spectrum.

Patient	Mean value	Standard deviation	Variation
Healthy	0.4265	0.0125	1.5703e−04
Celiac	0.3340	0.0354	0.0013

### Green spectrum variation

**Figure 15:**
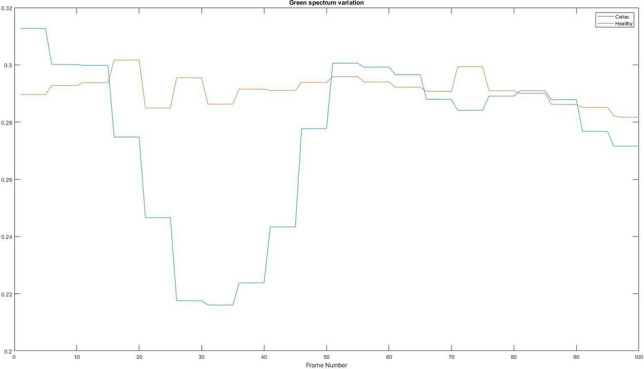
Green spectrum variation. Red = Healthy and Blue = Celiac.

The next spectrum to be analysed is the green spectrum, it is useful especially in frames where mud is present, because it is generally green for patients suffering from celiac disease. Figure 15 shows the variation of the green spectrum, highlighting the differences between a patient suffering from celiac disease and a healthy patient.

In Table 6 it is presented how the algorithm interprets these values using the mean value, standard deviation, and variation.

**Table 6:** Interpretation of the average variation of green spectrum.

Patient	Mean value	Standard deviation	Variation
Healthy	0.2914	0.0048	2.3215e−05
Celiac	0.2749	0.0289	8.3264e−04

### Blue spectrum variation

**Figure 16:**
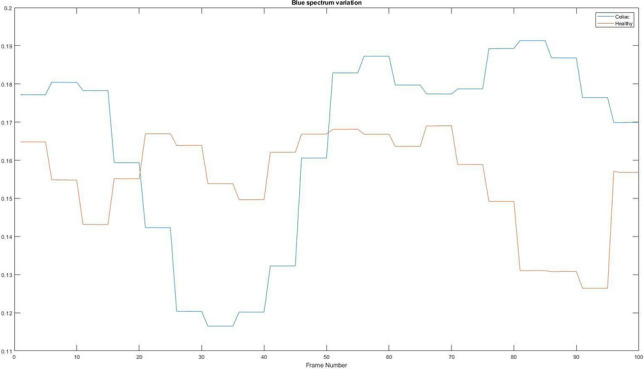
Blue spectrum variation. Red = Healthy and Blue = Celiac.

The last spectrum to be analysed is the blue one. With the help of the information of the three spectra, the algorithm can make a more concrete analysis of the images, managing to decompose it, obtaining four images (the three spectra plus the black and white image), each with different information. Figure 16 shows the variation of the blue spectrum showing the differences between a patient suffering from celiac disease and a healthy patient.

In Table 7 it is presented how the algorithm interprets these values using the mean value, standard deviation, and variation.

**Table 7:** Interpretation of the average variation of blue spectrum.

Patient	Mean value	Standard deviation	Variation
Healthy	0.1551	0.0129	1.6608e−04
Celiac	0.1654	0.0245	5.9790e−04

### The values obtained after applying the Sobel filter

**Figure 17:**
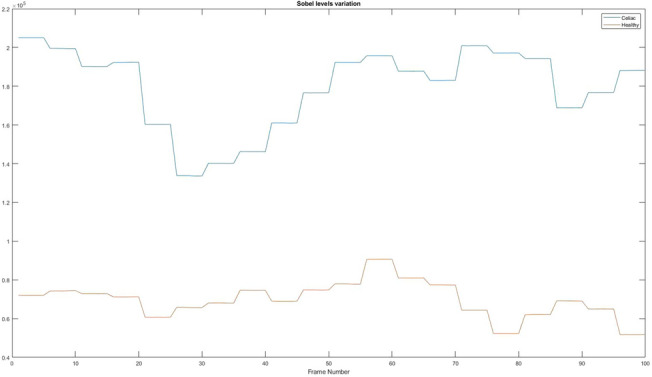
The values obtained after applying the Sobel filter. Red = Healthy and Blue = Celiac.

According to the algorithm presented in “Green spectrum variation” section, a binarization operation was applied to the obtained images, to be able to obtain only the values that are on the edges of the intestinal walls. Based on those values, a sum of the matrix pixels was made to be able to analyse the length of the folds with the help of a numerical value. So that in Fig. 17 you can see w the differences from a healthy patient to a sick one.

In Table 8 it is presented how the algorithm interprets these values using the mean value, standard deviation, and variation.

**Table 8:** Interpretation of the values obtained after applying the Sobel filter.

Patient	Mean value	Standard deviation	Variation
Healthy	6.9742e+ 04	9.0348e+03	8.1628e+07
Celiac	1.7951e+05	2.0710e+04	4.2892e+08

### The values obtained after applying the generated filter

**Figure 18:**
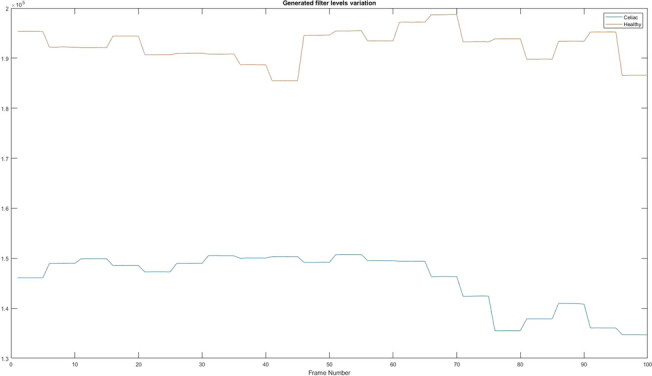
The values obtained after applying the generated filter. Red = Healthy and Blue = Celiac.

Based on the results obtained in “Blue spectrum variation” section, the algorithm makes a sum of pixels to determine the presence of cracks, but a high value present on the graphs indicates that there are not a large number of cracks, while a small value indicates a large number of cracks. Figure 18 shows the differences between a patient suffering from celiac disease and a healthy one.

In Table 9 it is presented how the algorithm interprets these values using the mean value, standard deviation, and variation.

**Table 9:** Interpretation of the values obtained after applying the Sobel filter.

Patient	Mean value	Standard deviation	Variation
Healthy	1.9261e+05	3.2661e+03	1.0668e+07
Celiac	1.4566e+05	5.4625e+03	2.9839e+07

### The value of entropy

**Figure 19:**
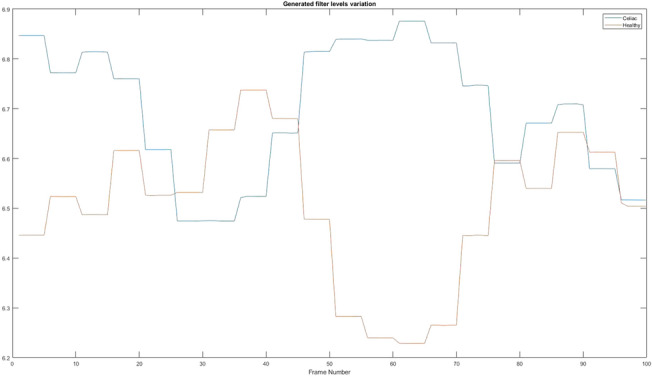
The value of entropy. Red = Healthy and Blue = Celiac.

With the help of entropy, the algorithm can analyse the texture of an image because it is calculated based on the histogram, as presented in “The values obtained after applying the Sobel filter” section. In Fig. 19 are presented the differences from a healthy patient to a sick one.

In Table 10 it is presented how the algorithm interprets these values using the mean value, standard deviation, and variation.

**Table 10:** Interpretation of the variation of entropy values.

Patient	Mean value	Standard deviation	Variation
Healthy	6.5025	0.1467	0.0215
Celiac	6.6973	0.1326	0.0176

### Variation in the number of large regions

**Figure 20:**
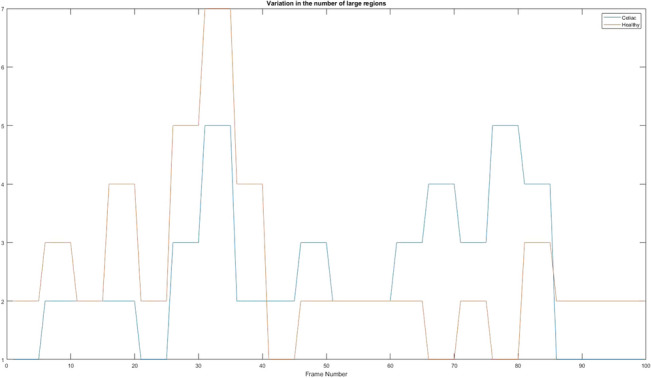
Variation in the number of large regions. Red = Healthy and Blue = Celiac.

After obtaining the images to which the Sobel filters were applied, the algorithm can identify certain continuous regions that generally represent the walls of the intestine between two folds. The first step to achieve this algorithm is to fill in each region, then it is necessary to find regions that have a larger area than the default (in the case of this application the value is 1000). In Fig. 20 can be observed the differences of regions from a patient suffering from celiac disease to a healthy patient. Even if at first impression the data do not seem to be very different, their ratio with those obtained from the application of the generated kernel is some of the best, because the differentiation between the data is easily observable.

In Table 11 it is presented how the algorithm interprets these values using the mean value, standard deviation, and variation.

**Table 11:** Interpretation of variation in the number of large regions.

Patient	Mean value	Standard deviation	Variation
Healthy	2.5500	1.4381	2.0682
Celiac	2.4500	1.2503	1.5631

### Variation in the number of small regions

**Figure 21:**
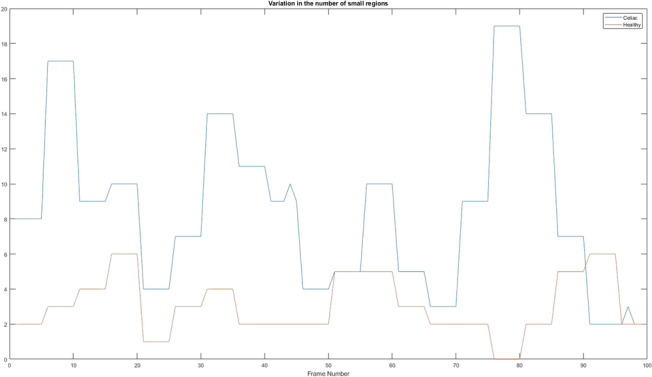
Variation in the number of small regions. Red = Healthy and Blue = Celiac.

After several analyses, the residual values remaining after searching for large regions can also be used to determine the existence of celiac disease. Figure 21 shows the differences from a healthy patient and a sick one.

In Table 12 it is presented how the algorithm interprets these values using the mean value, standard deviation, and variation.

**Table 12:** Interpretation of variation in the number of large regions.

Patient	Mean value	Standard deviation	Variation
Healthy	3.0500	1.6353	2.6742
Celiac	8.4700	4.6979	22.0698

### Obtained results

The obtained results, meaning the precision, sensibility and F1 value, are presented in Table 13.

**Table 13:** Performances of the used algorithms.

Used algorithm	Precision	Sensibility	F1 score
Fine KNN	0.84	0.8	0.81
Weighted KNN	0.92	0.93	0.92
Linear SVM	0.94	0.96	0.94

**Figure 22:**
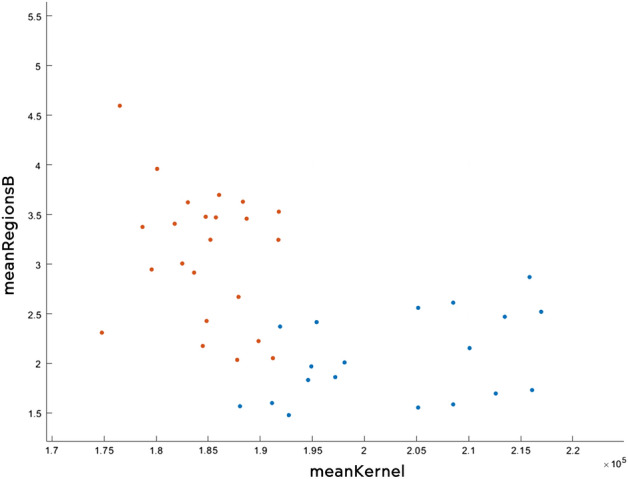
Correctly analysed data.

**Figure 23:**
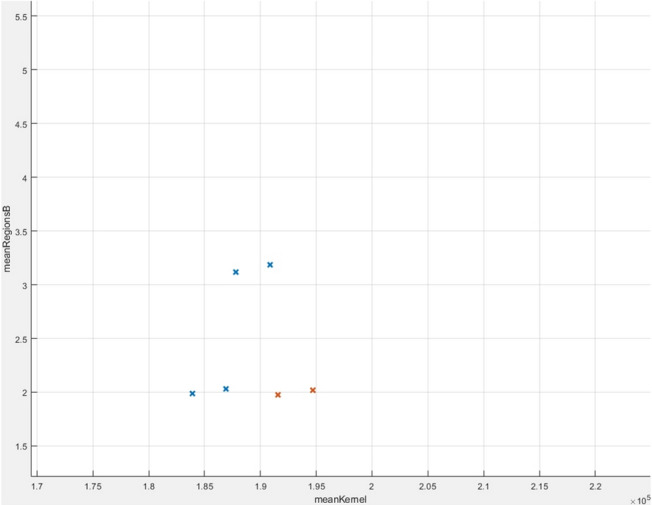
Wrong analysed data.

The algorithm fine KNN managed to obtain an accuracy of 84.3%, which means that out of 51 tests (30 sick and 21 healthy), 6 tests had an inadequate result. In Fig. 22 you can see all the data analysed correctly, while in Fig. 23 the wrong ones. The tests that were not correctly predicted are in the area where the two data sets meet, which can be offered by assessing the distances between neighbours.

**Figure 24:**
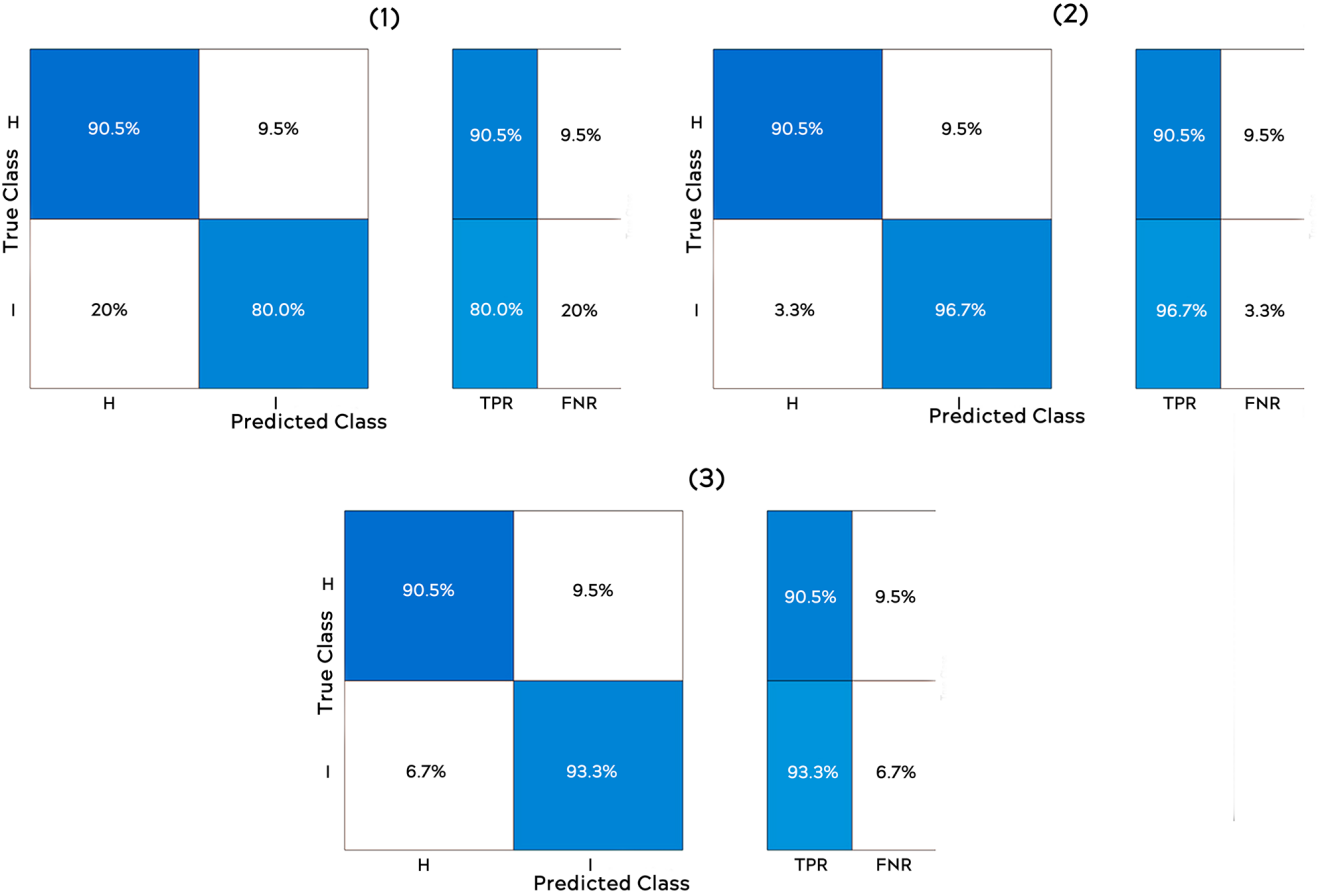
Confusion matrixes: (1) fine KNN; (2) weighted KNN; (3) linear SVM.

Figure 24 shows the confusion matrix for each method, from which can be observed the chances for a prediction to be true-positive or false-positive (same for the negative ones).

**Figure 25:**
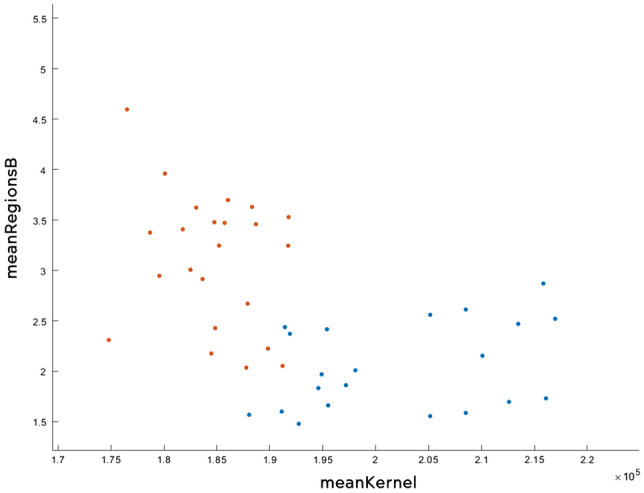
Correctly analysed data.

**Figure 26:**
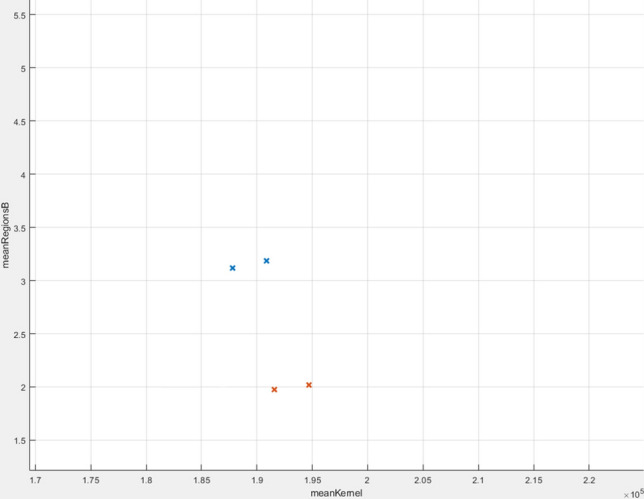
Wrong analysed data.

The algorithm Weighted KNN managed to obtain an accuracy of 92.2%, which means that out of 51 tests (30 sick and 21 healthy), 4 tests had an inadequate result. In Fig. 25 you can see all the data analysed correctly, and in Fig. 26 the wrong ones. The improvement of the result can be observed only by simply modifying the importance of the neighbours as presented in “The standard deviation of the light intensity value” section.

**Figure 27:**
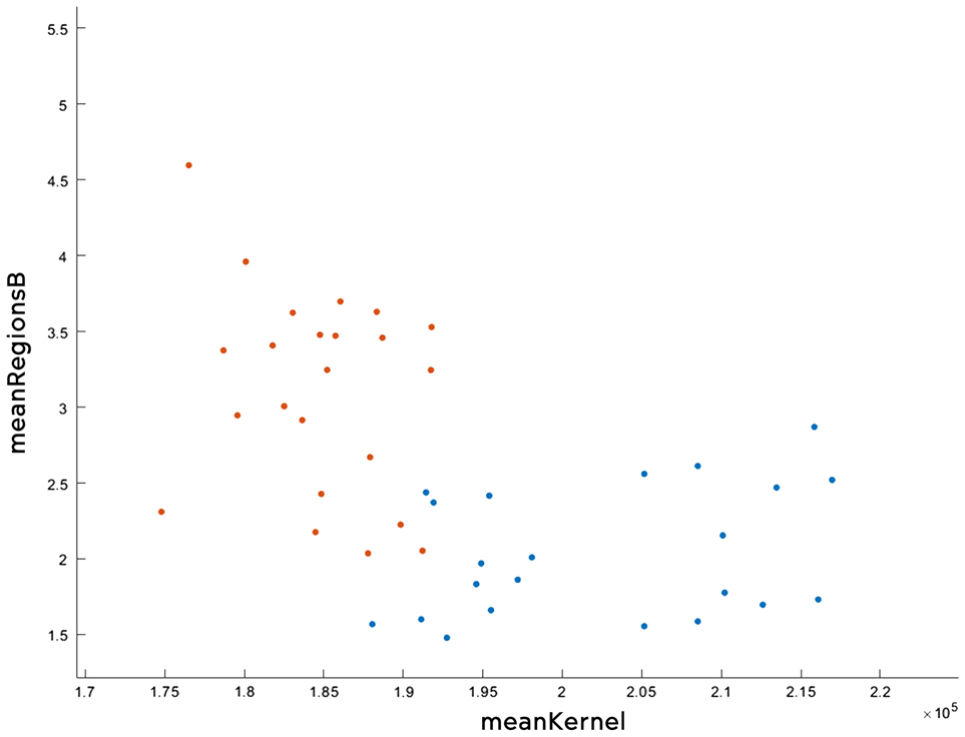
Correctly analysed data.

**Figure 28:**
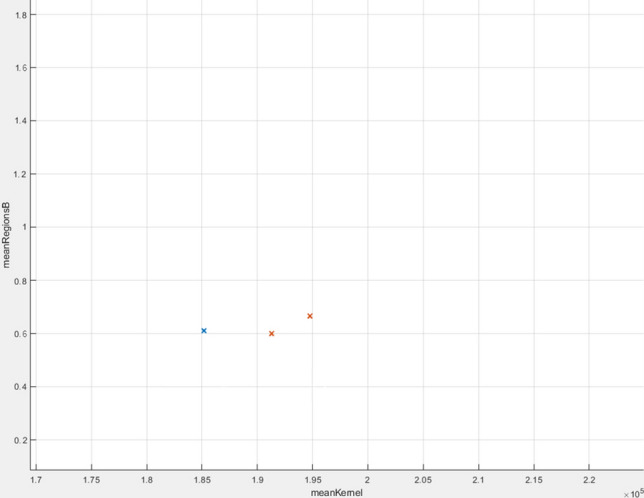
Wrong analysed data.

The linear SVM algorithm managed to obtain an accuracy of 94.1%, which means that out of 51 tests (30 sick and 21 healthy), 3 tests had an inadequate result. In Fig. 27 you can see all the data analysed correctly, while in Fig. 28 the wrong ones.

## Conclusion

Due to the limitation of the data set, it is easy to say that this study should be repeated with a greater sample size to verify if the results are correct and it should contain another type of data, the one that comes from patients with other diseases than celiac. But its main idea is to demonstrate that a diagnosis of celiac disease is possible with a computer, and it is not necessary to use very complex algorithms such as the references from Table 14. For this study the data set was formed by 109 videos (of 100 frames), of which 45 come from healthy patients and 65 come from patients suffering from celiac disease. The classification of these videos was realized using a PillCam SB3 capsule endoscopy device throughout the year 2019, with patients of different ages and genders, at Regional Institute of Gastroenterology and Hepatology, Cluj-Napoca, Romania.where the F1 score is:

**Table 14:** Final results of the Machine Learning Algorithms.

M.L. Algorithm	Accuracy	F1 score
Fine KNN	84.3%	81%
Weighted KNN	92.2%	92%
Linear SVM	94.1%	94%
Paper^5^	–	88%
Paper^11^	> 75%	
Paper^14^	93.84%	–
AlexNet^14^	90%	–
Paper^15^	93%	

4 $${F}_{1}=\frac{TruePozitive}{TruePozitive+\frac{1}{2}\left(FalsePozitive+FalseNegative\right)}.$$

Currently, there is much interest in incorporating quantitative technology for video endoscopy image analysis in the design of programs for celiac disease diagnosis and treatment. Endoscopy with histology assessment is the main tool in the diagnostic algorithm for celiac disease, but it is invasive and the diagnosis requires several days. The serological test for antibodies to tissue transglutaminase might be helpful, but false negative and false positive results should be considered. The use of automated programs for images acquired noninvasively through capsule endoscopy would be assistive to detect the subtle presence of villous atrophy not evident by visual inspection. It may also be useful to assess the degree of improvement of celiac patients on a gluten-free diet, the main treatment method for stopping the autoimmune process and improving the state of the small intestinal villi.

## Data Availability

The data that support the findings of this study are available on request from the corresponding author.

## References

[1] Popp A, Balaba VD, Mäki M, Gershman G, Thomson M (2021). Celiac disease. Practical Pediatric Gastrointestinal Endoscopy.

[2] Caio G (2019). Celiac disease: A comprehensive current review. BMC Med..

[3] Lewis NR, Scott BB (2006). Systematic review: The use of serology to exclude or diagnose coeliac disease (a comparison of the endomysial and tissue transglutaminase antibody tests). Aliment. Pharmacol. Ther..

[4] Cichewicz AB (2019). Diagnosis and treatment patterns in celiac disease. Dig. Dis. Sci..

[5] Wei JW (2019). Automated detection of celiac disease on duodenal biopsy slides: A deep learning approach. J. Pathol. Inform..

[6] Zhou T (2017). Quantitative analysis of patients with celiac disease by video capsule endoscopy: A deep learning method. Comput. Biol. Med..

[7] Koh JEW (2021). Automated interpretation of biopsy images for the detection of celiac disease using a machine learning approach. Comput. Methods Prog. Biomed..

[8] Shrivastava, A. *et al*. Deep learning for visual recognition of environmental enteropathy and celiac disease. In *2019 IEEE EMBS International Conference on Biomedical & Health Informatics (BHI)*, 1–4 (IEEE, 2019).

[9] Sali, R. *et al*. Celiacnet: Celiac disease severity diagnosis on duodenal histopathological images using deep residual networks. In *2019 IEEE International Conference on Bioinformatics and Biomedicine (BIBM)*, 962–967 (IEEE, 2019). 10.1109/bibm47256.2019.8983270 PMC8740775 35003830

[10] Hujoel IA (2018). Machine learning in detection of undiagnosed celiac disease. Clin. Gastroenterol. Hepatol..

[11] Piccialli F (2021). Precision medicine and machine learning towards the prediction of the outcome of potential celiac disease. Sci. Rep..

[12] Mehandiratta, A. *et al*. Prediction of celiac disease using machine-learning techniques. In *International Conference on Innovative Computing and Communications*, 663–673 (Springer, 2020).

[13] Ciaccio EJ (2010). Classification of videocapsule endoscopy image patterns: Comparative analysis between patients with celiac disease and normal individuals. Biomed. Eng. Online.

[14] El-Matary W, Huynh H, Vandermeer B (2009). Diagnostic characteristics of given video capsule endoscopy in diagnosis of celiac disease: A meta-analysis. J. Laparoendosc. Adv. Surg. Tech..

[15] Kowsari, K. Diagnosis and analysis of celiac disease and environmental enteropathy on biopsy images using deep learning approaches. Preprint at http://arXiv.org/2006.06627 (2020). 10.1007/978-3-030-32520-6_55 PMC8536862 34726364

[16] Fisher, R. *et al*. A to Z of image processing concepts. In *Convolution and Kernels* (2000). http://homepages.inf.ed.ac.uk/rbf/HIPR2/kernel.htm. Accessed June 2021.

[17] Zhao S (2021). Multilevel threshold image segmentation with diffusion association slime mould algorithm and Renyi’s entropy for chronic obstructive pulmonary disease. Comput. Biol. Med..

[18] Memon, N., Patel, S. B. & Patel, D. P. A novel approach of polsar image classification using Naïve Bayes classifier. In *Mathematical Modeling, Computational Intelligence Techniques and Renewable Energy: Proceedings of the First International Conference, MMCITRE 2020*, 93–104 (Springer, 2021).

[19] Shokrzade A (2021). A novel extreme learning machine based kNN classification method for dealing with big data. Expert Syst. Appl..

[20] Abdelsalam MM, Zahran MA (2021). A novel approach of diabetic retinopathy early detection based on multifractal geometry analysis for OCTA macular images using support vector machine. IEEE Access..

